# Radiation Treatment in Women with Ovarian Cancer: Past, Present, and Future

**DOI:** 10.3389/fonc.2017.00177

**Published:** 2017-08-21

**Authors:** Emma C. Fields, William P. McGuire, Lilie Lin, Sarah M. Temkin

**Affiliations:** ^1^Division of Radiation Oncology, Virginia Commonwealth University, Richmond, VA, United States; ^2^Internal Medicine, Virginia Commonwealth University, Richmond VA, United States; ^3^Division of Radiation Oncology, University of Texas, MD Anderson Cancer Center, Houston, TX, United States; ^4^Division of Gynecologic Oncology, Virginia Commonwealth University, Main Hospital, Richmond, VA, United States

**Keywords:** ovarian cancer, radiation, novel therapeutics, poly(ADP-ribose) polymerase inhibitors, abscopal effect

## Abstract

Ovarian cancer is the most lethal of the gynecologic cancers, with 5-year survival rates less than 50%. Most women present with advanced stage disease as the pattern of spread is typically with dissemination of malignancy throughout the peritoneal cavity prior to development of any symptoms. Prior to the advent of platinum-based chemotherapy, radiotherapy was used as adjuvant therapy to sterilize micrometastatic disease. The evolution of radiotherapy is detailed in this review, which establishes radiotherapy as an effective therapy for women with micrometastatic disease in the peritoneal cavity after surgery, ovarian clear cell carcinoma, focal metastatic disease, and for palliation of advanced disease. However, with older techniques, the toxicity of whole abdominal radiotherapy and the advancement of systemic therapies have limited the use of radiotherapy in this disease. With newer radiotherapy techniques, including intensity-modulated radiotherapy (IMRT), stereotactic body radiotherapy (SBRT), and low-dose hyperfractionation in combination with targeted agents, radiotherapy could be reconsidered as part of the standard management for this deadly disease.

## Introduction

Epithelial ovarian cancer is the most lethal of the gynecologic cancers, with a 5-year survival rate less than 50%. Each year, an estimated 22,000 women will be diagnosed with and 14,000 women will die from the disease (1). The emergence of molecular and genetic data over the past decade has improved the understanding of the heterogeneity of ovarian cancer. Type I tumors consist of low-grade serous, low-grade endometrioid, clear cell carcinomas, and mucinous carcinomas and are characterized by mutations in *KRAS, BRAF, PTEN, PIK3CA, CTNNB1, ARID1A*, and *PPP2R1A*. These cancers tend to be diagnosed while confined to the ovary and are relatively chemotherapy resistant. Type II ovarian cancers are the more common of the ovarian cancer histotypes, consisting of high-grade serous (70%), high-grade endometrioid, carcinosarcoma, and undifferentiated carcinomas. Type II tumors are defined by *TP53* mutations, which are rare in Type I cancers (2–5). Women diagnosed with Type II cancers typically present with few or vague symptoms. As a result, the majority of women present with Stage III or IV disease where disease is present in the upper abdomen or outside of the peritoneal cavity or within hepatic parenchyma.

With aggressive therapy at diagnosis, including surgery and platinum-based chemotherapy, more than 80% of women diagnosed with advanced disease will have an initial complete response. Unfortunately, these responses are infrequently durable and the majority of women with ovarian cancer develop recurrent, disease, which is typically incurable although subsequent response and months of survival may still be possible. Unfortunately, responses to subsequent chemotherapeutic regimens shorten in duration over time due to progressive development of resistance to platinum-based chemotherapy. Novel treatment strategies are urgently needed in order to improve survival.

Despite evidence that ovarian cancer is a radiosensitive malignancy, the use of radiation as a therapeutic modality in the modern era is limited (6–10). Ovarian cancer has a unique pattern of dissemination as transperitoneal spread is the most common route such that, diagnosis, the tumor is confined to the abdominal and pelvic cavity in approximately 85% of patients. Adjuvant radiation therapy was historically used in the adjuvant setting for the management of ovarian carcinoma of all tumor subtypes with reasonable results (6, 10). Because ovarian cancer is rarely confined to the pelvis, whole pelvic radiation is a largely ineffective method of disease control since it does not treat the entire volume at risk of recurrence. Whole abdominal radiotherapy (WAR) was used in the pre-chemotherapy era to sterilize large volumes of micrometastatic intraperitoneal disease. However, the low doses required to meet tolerance of the bowel, kidneys, and liver using two dimensional fields were ineffective in eradicating gross residual disease in the peritoneal cavity resulting in poor therapeutic efficacy. Additionally, the toxicity of radiation therapy was high particularly when using wide-field irradiation. High rates of both acute and late toxicity, particularly gastrointestinal, resulted in the abandonment of radiation in this disease particularly when cisplatin was confirmed to be a highly active systemic agent. Improved radiation techniques with lower toxicity have led to a renewed interest in the use of radiation therapy for metastatic cancers for many disease sites including ovarian cancer. In this article, we summarize the historical use of radiotherapy for ovarian cancer and discuss its potential role in the era of intensity-modulated radiotherapy (IMRT) and image guided radiotherapy as well as its integration with novel therapies.

## Intraperitoneal P32

In a colloidal suspension form, phosphorus-32 (^32^P) forms a complex, insoluble particle, which can be injected directly into the peritoneal cavity. The colloidal suspension then prevents radioisotope from leaving the intended target and disseminating throughout the body (11, 12). The first reported clinical application of ^32^P was in the 1930s and other intraperitoneal radioactive isotypes were investigated in the 1950s and 1960s most notably ^198^Au, but for reasons of safety and toxicity, ^32^P became the agent of choice for the treatment of ovarian cancer and the palliation of malignant ascites in the 1960s (12, 13). Many reports of therapeutic ^32^P were encouraging (13–16).

### Advanced Disease

For patients with advanced disease, ^32^P combined with WAR was investigated but found to be overly toxic. In a review of 95 patients using ^32^P for ovarian cancer, Tharp and Hornback demonstrated that the 5-year chronic complication rates (predominately gastrointestinal) were 44% when adjunctive pelvic or whole abdominal radiation was added compared with 17% (5% if minor complaints excluded) if used alone (*p* = 0.04) (17). Klaassen and colleagues from the National Cancer Institute of Canada (NCIC), similarly abandoned further development of ^32^P combined with pelvic radiotherapy in the treatment of high-risk early stage ovarian cancer due to excessive toxicity (18). Nevertheless, ^32^P continued to be investigated as consolidation therapy for patients with advanced disease after pathologically documented (by second look laparotomy) complete remission following primary surgery and platinum-based surgery. A Gynecologic Oncology Group (GOG) study enrolled 202 patients who were randomized to ^32^P vs. no further treatment following a negative second look surgery. No difference in relapse rates or survival was observed and toxicity was higher in the treatment arm (19).

### Early Stage Disease

A large prospective clinical trial to assess the utility of ^32^P in early stage ovarian cancer was opened in 1976 by the Ovarian Cancer Study Group and joined in 1978 by the GOG. Patients with Stage I, Grade 3 or Stage II disease were randomly assigned to receive either melphalan or intraperitoneal ^32^P [15 mCi of chromic phosphate (before 1979 the dose was 7.5 mCi)] as primary postoperative adjuvant therapy. One hundred forty-five patients were randomly assigned, 71 to receive melphalan and 74 to receive ^32^P. The study was closed to new patients in November 1986 and no survival differences were seen in either arm (20). Criticisms of this trial include the inclusion of suboptimally staged patients (nodal sampling was not required) and patients with tumors of low malignant potential (22%). Toxicity differences between the arms included increased late gastrointestianl toxicity in the ^32^P arm and secondary hematologic malignancies in the melphalan arm. Despite these limitations, three subsequent prospective trials compared intraperitoneal ^32^P to adjuvant chemotherapy in patients with early stage disease (20–23). Although survival was similar between arms, ^32^P for early stage disease was abandoned due to the easier administration and lower toxicity of newer, improved systemic agents (platinum). An improved emphasis on surgical staging and a better understanding of the histologic subtypes of ovarian cancer led to better definitions of early stage patients in whom adjuvant therapy could be omitted in favor of close observation (24).

## Whole-Abdomen Radiation Therapy

**Figure 1:**
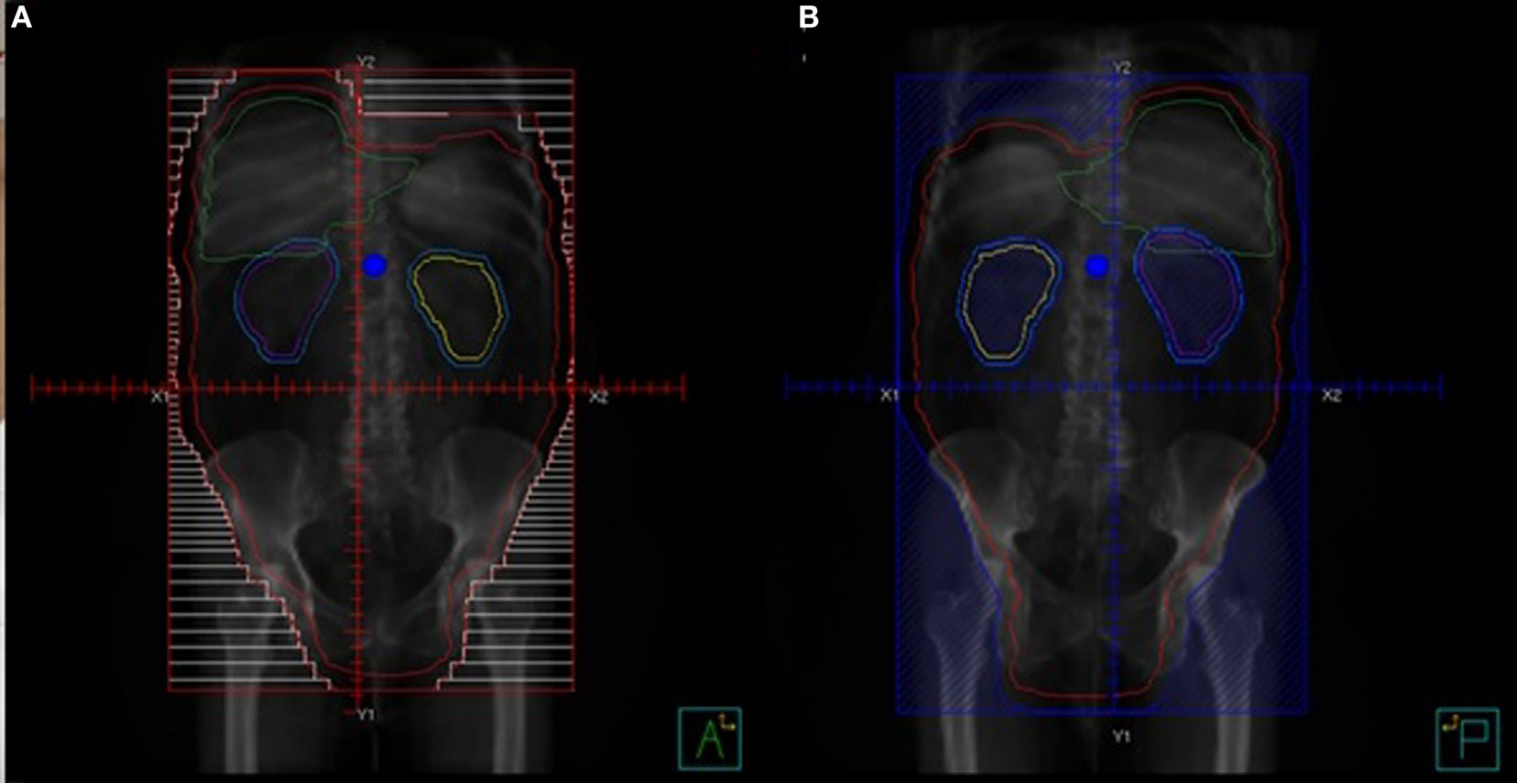
Techniques for treating whole abdominal radiation. **(A)** Open field technique AP beam. **(B)** Open field technique PA beam with kidney blocks.

In the 1960s, a “moving strip” technique for the delivery of WAR was developed. Because available technology could not adequately deliver radiation to the entire abdomen, the patient’s abdomen was marked with 12–14 “strips,” each 2.5 cm in height, and treated for approximately 10 weeks. With daily fractions of 225–300 cGy, a cumulative dose of 2,250–3,000 cGy was able to be delivered (Figure 1A) (25, 26).

The development of open-field techniques as an alternative to the moving strip technique allowed daily treatment with shorter duration courses of radiation delivery. Both techniques have been compared prospectively for efficacy and toxicity and found to be similar (27, 28). Because the open field technique was associated with fewer serious late toxicities, is simpler to plan, and takes less time to administer, it became the favorable technique (Figures 1A,B) (26, 28).

### Adjuvant WAR following Surgery

Several prospective trials conducted in the 1970s showed promising results of postoperative radiation following surgery (Table 1) (25, 29–35). Although patients with bulky disease following surgery were rarely cured, those patients with microscopic residual disease after initial cytoreductive surgery experienced disease-free survivals of 42–62% at 10 years in two randomized trials (29, 30). The first of these trials randomized 149 patients with early ovarian cancer comparing WAR with additional irradiation to the pelvis and melphalan. Although improved survival was not demonstrated in the study population, a benefit for Stage I patients who received WAR was seen (29). A second study of pelvic radiation plus WAR compared to pelvic radiation plus chlorambucil did demonstrate an overall survival benefit (78 vs. 51%; *p* = 0.006) (30). The combined results of these two studies were used to justify the development of a risk model on the basis of stage, grade, and amount of residual disease to define categories of patients most likely to benefit from WAR (10, 26). Ideal patients for WAR were thought to be Stage I, Grade 2 or 3; Stage 2 disease except Grade 3 patients with residual disease; and Stage III, Grade 1 patients with <2 cm residual disease (26).

**Table 1:** Studies comparing whole abdominal radiotherapy (WAR) vs. chemotherapy.

Study	*N*	Inclusion criteria	Treatment arms	5-year survival rates	
				WAR	CHEMO (%)
**Adjuvant radiotherapy**					
MDACC Smith et al. (29)	149	Stage I–III	WAR vs. melphalan	71%	72

Gynecologic Oncology Group Hreshchyshyn et al. (36)	86	Stage I	No further therapy vs. WAR vs. melphalan	70%^a^ (*p* < 0.05)	94

Princess Margaret Dembo et al. (30)	147	Stage I–III	PR + WAR vs. PR + cholorambucil	58%^a^ (*p* < 0.05)	41

National Cancer Institute of Canada Klaassen et al. (31)	257	Stage I–IIIA	Arm A: WAR Arm B: Melphalan + pelvic RT Arm C: ^32^P + pelvic RT (dropped due to toxicity)	61%	62

Denmark Sell et al. (33)	118	Stage IB–II	WAR vs. cyclophosphamide + pelvic RT	55%	63

West Midlands I Redman et al. (32)	40	Stage IC–III, no residuum	WAR vs. 5 cycles cisplatin	58%	62

National Cancer Institute (NCI) Italy Chiara et al. (34)	70	Stage I–III	WAR vs. 6 cycles cisplatin + cyclophosphamide	53%	71

French Kojs et al. (35)	150	Stage IA, IB (G2–3), IC, IIA no residuum	WAR vs. cisplatin, adriamycin, cyclophosphamide	81%	81

**Consolidation radiotherapy**					

West Midlands II Lawton et al. (37)	109	Surgery → cisplatin × 5 → second look surgery	Arm A: WAR Arm B: chlorambucil × 1 year	35%^b^	35^b^

British Columbia Hoskins et al. (38)	131	Stage IG3, IIG3, III no residuum	Arm A (1983–1989): cisplatin + cyclophosphamide × 6 cycles with WART between cycles 3 and 4 Arm B (1989–1991): cisplatin × 6 cycles	78%	78

North Thames Ovary Study Group Lambert et al. (39)	117	Stage IIB–IV ≤2 cm residuum	Arm A: WAR Arm B: carboplatin	32%	32

Austrian Pickel et al. (40)	32	Stage IC–IV → carboplatin, epirubicin, prednimustine × 6	Arm: A: WAR Arm B: observation	59%^a^ (*p* < 0.05)	33

Swedish Sorbe and Swedish-Norgewian Ovarian Cancer Study Group (41)	172	III no or microscopic residuum (*n* = 98)	Arm A: WAR Arm B: chemotherapy Arm C: no further therapy	69%	57
		Macroscopic disease	Arm A: WAR Arm B: chemotherapy	32%	41

*^a^10 year survival*.

*^b^2 year survival rates*.

Enthusiasm for this risk model was tempered by the results of a multicenter trial of 257 patients with Stage I, II, or IIIA disease who were randomized to WAR or melphalan or ^32^P following whole pelvic radiation. The combination of pelvic radiation therapy plus ^32^P was exceedingly toxic, and this arm was dropped after 44 patients were enrolled. Patients who received melphalan had improved disease-free survival but overall survival was similar between all three arms (31). Criticisms of this study included the number of inadequately surgically staged patients, a large number of protocol violations and the excessive radiation related toxicity.

Additional prospective trials of WAR vs. chemotherapy are included in Table 1. Radiation began to fall out of favor at this time as an adjuvant treatment modality due to the additional toxicity of WAR compared to chemotherapy, the complexities of radiation delivery compared to chemotherapy, and the advent of more effective cytotoxic agents for the treatment of ovarian cancer.

### Consolidation Therapy (following Surgery and Chemotherapy)

During this era of ovarian cancer treatment, when the benefits of radiation were being evaluated, second look laparotomy was routinely performed after completion of primary therapy in ovarian cancer when imaging and markers suggested a complete clinical response. Patients with minimal residual disease at the time of second look surgery seemed most likely to have improved survival from WAR, and there were reports of long-term survivors in this subset of patients. Despite concerns about toxicity, the use of radiation as consolidation following surgery and chemotherapy continued to be explored (38, 42–46). Retrospective investigations demonstrated 3-year progression-free survival rates of 50–67% (43, 44) and 5-year overall survival rates of 40–66% (45, 46).

The largest of the prospective trials examining consolidation WAR are presented in Table 1. The West Midlands Ovarian Cancer Group Trial II reported a prospective randomized comparison of WAR vs. 1 year of chlorambucil treatment. The randomization was done after primary surgery, chemotherapy, and second look surgery. Fifty-six patients were randomly assigned to receive WAR and 53 patients received chlorambucil after second look surgery. Methodologic problems as well as slow accrual make the results difficult to interpret, but survival was not statistically different between treatments (32). A subsequent Canadian trial compared chemotherapy alone to 3 cycles of cyclophosphamide and cisplatin followed by WAR followed by three additional cycles of chemotherapy. Survival was improved for the subset of patients with Stage I disease although overall 5-year survival was equivalent in both arms (38). A prospective trial from the North Thames Ovary Study Group, randomized 117 patients with residual disease of 2 cm or less at second-look laparotomy or laparoscopy to receive consolidation therapy, with either additional carboplatin or WAR (24 Gy) with no difference in survival seen between arms but toxicity favored the chemotherapy arm (39). Additionally, a Swedish prospective study randomized 172 patients with stage III ovarian cancer to receive consolidative whole abdominal radiotherapy, further chemotherapy, or no additional therapy following second look laparotomy. For patients with pathologic CR (*n* = 98), there was significantly improved progression-free survival (56 vs. 36%) and improved overall survival (69 vs. 57%) favoring WAR (*p* = 0.032). In patient with microscopic residual disease (*n* = 74), there was no difference in progression free or overall survival between the two arms. No survival difference was seen in those patients with macroscopic disease. Late, grade 3 bowel toxicity was reported in 10% of patients (41). The only truly positive study examining WAR consolidation was an Austrian study with long-term follow-up which showed a relapse-free survival and overall survival benefit for WAR compared to observation after a clinical complete response to six cycles of triplet chemotherapy. This benefit was most pronounced in women with stage III disease with a relapse-free survival at 5 years of 45 vs. 19% (*p* = 0.0061) and overall survival at 5 years of 59 vs. 26% (*p* = 0.012) (40).

A recent Surveillance, Epidemiology, and End Results Program analysis demonstrated an overall worse survival for patients who received RT for ovarian cancer. Yet, interestingly, the subset of patients with Stage III disease who received RT had a better OS at 5 years (54 vs. 44%) and 10 years (36 vs. 30%, *p* = 0.037) (47) compared to those who did not receive radiation. These results should be interpreted with caution due to the limitations of this database but provide continued support for some therapeutic role of radiation in a subset of ovarian cancer patients.

### Clear Cell Histology

Ovarian clear cell carcinoma has discrete clinical and molecular characteristics compared with the most common serous histologic type (48). Part of the interest in using radiation in clear cell carcinoma of the ovary is the relative chemotherapeutic resistance of this histogenetic type of ovarian cancer compared to the more common serous histopathology (48). A population-based study from British Columbia, Canada suggested that WAR provides a survival benefit when added to chemotherapy particularly in low-stage disease. In this report, using retrospective data, patients with Stage IC ovarian clear cell carcinoma treated with carboplatin and paclitaxel followed by abdominopelvic radiation had statistically superior disease-free survival compared to patients who had no radiation after initial chemotherapy. The absolute increase in disease-free survival following radiation was 20% at 5 years (49). Nagai demonstrated a survival advantage for adjuvant radiation in 16 ovarian clear cell carcinoma patients, stage IC-III treated with WAR in comparison to 12 patients from a historical cohort that received postsurgical platinum-based chemotherapy (50). The improved progression-free survival correlated to a significantly improved 5-year overall survival in the RT group of 81.8 vs. 33.3%. The methodological problems with this study include the small sample size, the comparator group of historic controls, as well as the potential selection bias in the prospective cohort. However, a very large retrospective Canadian study of 700 ovarian cancer patients treated with platinum-based chemotherapy alone or combination platinum-based chemotherapy combined with WAR, demonstrated a benefit from radiation for women with low-stage, microscopic residual disease, and non-serous histology—a 40% reduction in disease specific mortality at 10 years was observed (51). Conversely, a more recent Canadian retrospective review of 163 patients with early stage ovarian clear cell carcinoma treated at two institutions over a 20-year time period with or without adjuvant pelvic and/or WAR was not able to demonstrate a progression free or overall survival benefit (52). Furthermore, the most common patterns of failure were multifocal and distant, limiting the value of either pelvic radiation or WAR (53). These results in composite suggest a potential role for radiation therapy in ovarian cancer patients with low-stage clear cell histology; however, further research is needed to determine the most appropriate therapy for this group of patients.

### Toxicity of WAR—Why WAR Fell Out of Favor

Despite the perceived benefits of radiotherapy in intermediate and high-risk epithelial ovarian cancer as well as clear cell ovarian cancer, WAR has largely fallen out of the standard treatment paradigm for ovarian cancer. Indeed, the toxicity of WAR was significant—few patients made it through a treatment course without side effects, some significant enough to halt completion of therapy. During treatment, acute toxicity included diarrhea, fatigue, nausea, and hematologic effects. More concerning, however, were the long-term toxicities, which included basal pneumonitis in up to 20% of patients, liver damage, and bowel toxicity (10–15% of patients). Among 1,098 patients studied prospectively with WAR as ovarian cancer treatment, 5.6% underwent surgery for bowel obstruction, and there were four deaths related to bowel toxicity from radiation treatment (26). In addition to toxicity, the era of aggressive cytoreduction and effective chemotherapy (platinum agents) called into question the therapeutic index of radiation in the treatment of ovarian cancer.

## Salvage RT and Stereotactic Body Radiotherapy (SBRT)

Radiation therapy for ovarian cancer has, therefore, been predominantly confined to the palliative setting, used for symptom control, or to treat localized metastatic disease. Several retrospective studies have demonstrated the utility of targeted involved-field radiotherapy to doses of 45–60 Gy given in 1.8–2 Gy fractions (54–57). For patients with solitary recurrences, excellent local control (89–100%) has been shown (55, 57). Despite being heavily pretreated with chemotherapy, similar rates of progression-free survival and statistically longer rates of median overall survival compared to a similar group of women who received salvage chemotherapy at the time of recurrence have been described (55, 56). Although thought provoking, these studies deserve caution as the sample sizes are small, a selection bias exists for patients treated with this modality and the patients in comparison arms may not have received contemporary chemotherapy regimens. With minimal long-term toxicities, involved-field radiotherapy allows for longer chemotherapy-free intervals and should be considered as part of the treatment paradigm for salvage therapy of isolated recurrences (54).

**Figure 2:**
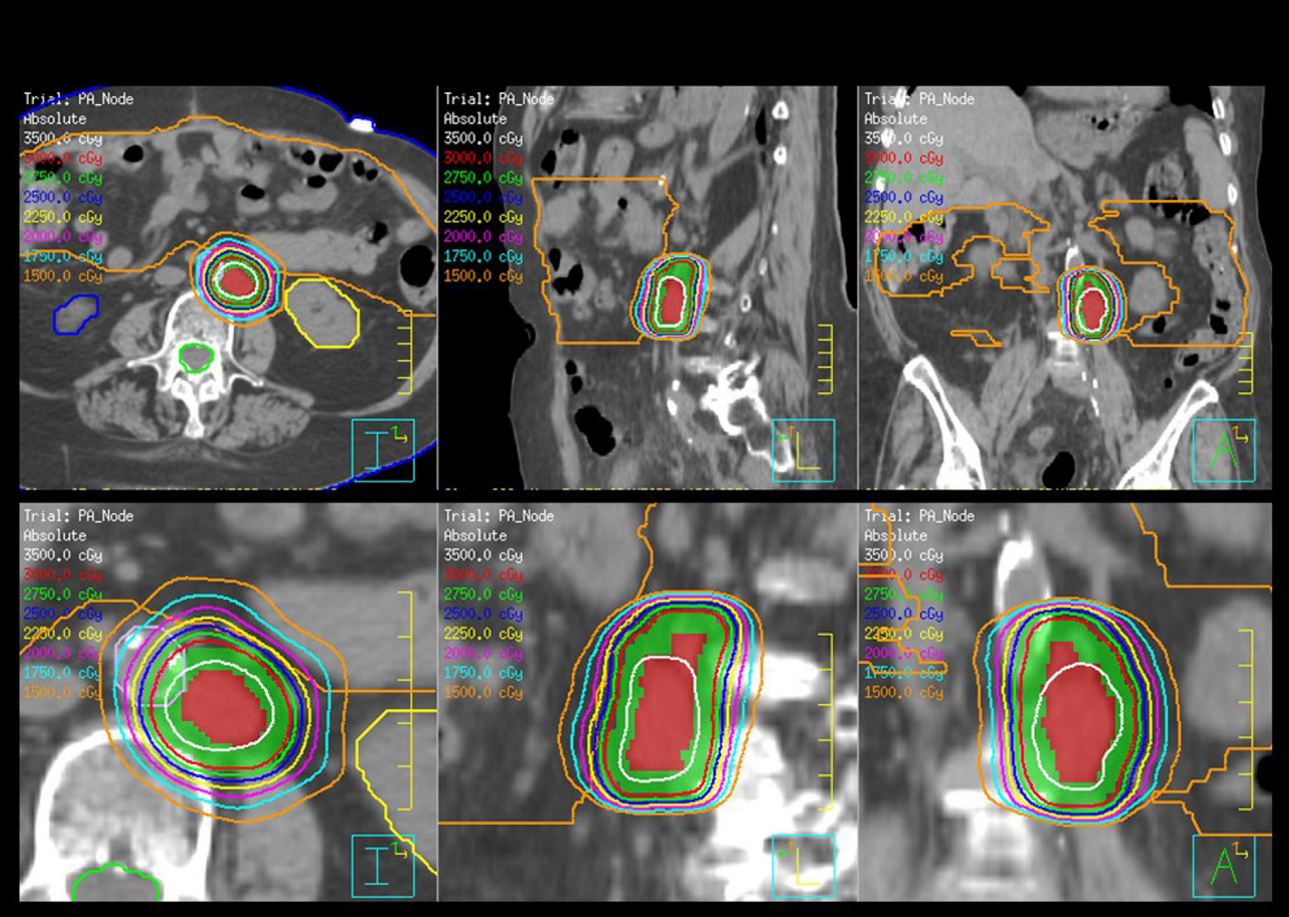
Stereotactic body radiotherapy (SBRT) plan for the treatment of a para-aortic lymph node for a woman with recurrent serous ovarian cancer. The plan is shown in axial, sagittal, and coronal orientations. The planning target volume is color washed in green, the kidneys are yellow, and blue and the bowel is orange. The dose is 30 Gy and prescribed in five fractions.

Stereotactic body radiotherapy (SBRT) is one of the newer options for palliative or salvage radiotherapy that allows for focused high-dose radiotherapy to the tumor with minimal dose to organs in close proximity and is ideal for residual or limited metastatic disease. SBRT is linear accelerator-based focal radiotherapy delivered with rigid patient and tumor immobilization, elegant dosimetry, and daily image guidance for verification of set up. SBRT implies a high-dose per fraction and is delivered in 2–5 fractions. SBRT serves to decrease tumor burden, destroy chemoresistant tumor clones, and help stimulate innate immune response or expose tumor neoantigens, providing excellent rates of local control, minimal acute and late toxicities, and can be used in women who have had prior radiotherapy (58–60). Figure 2 shows an SBRT plan treating a metastatic para-aortic lymph node in women with serous ovarian carcinoma who had previously received multiple lines of systemic therapy.

Multiple studies of SBRT in the management of metastatic gynecologic malignancies have been published (Table 2) (61–64). In a Phase II study, 50 women (50% with primary ovarian cancer) with ≤4 sites of metastatic disease were treated with SBRT to a dose 8 Gy × 3 fractions using Cyberknife. Common treatment sites included para-aortic nodes (38%), pelvic nodes (28%), and the liver (16%). The clinical target volumes were delineated based on co-registration to PET/CT imaging and 3 mm were added to create the planning target volume, which is a margin on the clinical target volume to account for set-up uncertainties and organ motion. Treatments were prescribed to the 70% isodose line and delivered in three consecutive days. Target response rate (defined as complete or partial response) was 96% with duration of follow-up of 6 months. Median disease-free survival was 7.8 months and overall survival was 20.2 months with only 3 grade ≥3 toxicities. These data are in line with results of salvage cytotoxic therapy in similar patients and the treatment is less intrusive for patients with limited survival.

**Table 2:** Stereotactic body radiotherapy for recurrent/metastatic ovarian cancer.

Study	*N*	Inclusion criteria	SBRT dose	Local control	Distant progression
Phase II Cleveland SBRT trial Kunos et al. (61)	50 pts (103 lesions)	≤4 metastatic sites, ovarian, cervical, endometrial cancers	8 Gy × 3 fractions daily	96% at 6 months	62% at 6 months

Italy Deodato et al. (65)	11 pts (12 lesions)	Confirmed recurrent/metastatic ovarian, cervical, endometrial cancers	6 Gy × 5 fractions daily	66.6% at 2 years	46% at 2 years

UNC Higginson et al. (66)	16 patients	Pelvic, PA nodes, metastatic disease, or substitute for brachytherapy for ovarian, vaginal, cervical, and endometrial cancers	12–54 Gy in 3–5 fractions	79% at 2 years	43% at 2 years

University of California Mesko et al. (67)	28 patients (47 lesions)	Confirmed recurrent/metastatic ovarian, vaginal, cervical, endometrial cancers	Median of 8 Gy × 5 fractions	34% stable disease, 32% partial response and 17% complete response at 1 year	57% at 1 year

Phase I Ohio Kunos et al. (68)	12 (28 lesions)	≤4 metastatic sites, ovarian, primary peritoneal, endometrial cancers	Carboplatin + gemcitabine + SBRT to 8Gy × 3 fractions	79% partial response, 21% stable disease at 6 weeks	75% at 6 weeks

Despite excellent local control, rates of progression outside of the targeted lesions remain high, ranging from 43 to 57% (65–67). These high rates of distant progression prompted a Phase I study with 12 women (7 with primary ovarian cancer) testing the safety of sequential carboplatin and gemcitabine followed by SBRT. This study showed that carboplatin AUC 4 and gemcitabine 600 mg/m^2^ can be given the day prior to SBRT using Cyberknife with the 8 Gy × 3 fraction regimen with acceptable rates of toxicity. These reassuring results may pave the way for the addition of radiosensitizers, targeted agents, and immunotherapy in combination with SBRT (69–72).

## The Future of Radiation Therapy in Ovarian Cancer

Many of the toxicities of WAR in ovarian cancer are due to the large volume of tissue receiving a high dose of radiotherapy with little sparing of the organs at risk as well as minimal time for intrafraction repair of normal tissues. Over recent years, newer, more palatable fractionation schemes and advanced techniques, which allow sparing of at risk organs may allow for renewed interest in this treatment modality for this disease. Improved radiation techniques combined with an increasingly sophisticated understanding of molecular mechanisms leading to radiation and chemotherapy sensitivity are leading to innovative and novel therapies for patients with ovarian cancer.

### Intensity-Modulated Radiotherapy

IMRT has replaced three dimensional conformal radiotherapy in the treatment of complex tumors, which are in close proximity to organs at risks such as in the treatment of prostate cancer, anal cancer, and head and neck malignancies. IMRT uses a computer algorithm to optimize dose to the target and minimize dose to organs at risk by modulating and shaping the beam either with static or dynamic beams. Rochet and colleagues examined the practicality of whole abdominal IMRT after surgical cytoreduction and chemotherapy in patients with advanced ovarian cancer. In the Phase I portion of the study (OVAR-IMRT-01), they treated 16 women (10 on study and 6 per protocol) with stage III disease (*n* = 15 had IIIC) (73). All women received optimal primary resection with <1 cm residual disease followed by six cycles of carboplatin and docetaxel. The radiotherapy was delivered with either static beams or helical tomotherapy and was delivered to a dose of 30 Gy in 20 fractions (74). In order to accurately deliver conformal treatment, women were immobilized with vacuum bags and masks. The clinical target volume included the entire peritoneal cavity from the diaphragm to the Douglas cavity as well as the pelvic and para-aortic lymph nodes. The planning target volume was a 1.5 cm axial expansion and 2.5 cm superior–inferior expansion with 1 cm expansion into the liver, but no additional margin on the kidneys. In their study, all patients completed planned treatments with no interruptions and no acute grade 4 toxicities. Results are promising—with 4 years of follow-up, there have been relatively low rates of early severe toxicity although late toxicities include six small bowel obstructions, three due to adhesions and three from tumor recurrence. The recurrence-free survival was 27.6 months and median overall survival 42.1 months (75).

These results prompted the OVAR-IMRT-02 study—an ongoing single-center one arm phase-II trial. Thirty seven patients with optimally cytoreduced stage III ovarian cancer with a clinical complete remission after chemotherapy will be treated with intensity-modulated WAR as consolidation therapy (76). The primary endpoint is tolerability and the secondary objectives are toxicity, quality of life, progression free, and overall survival.

**Figure 3:**
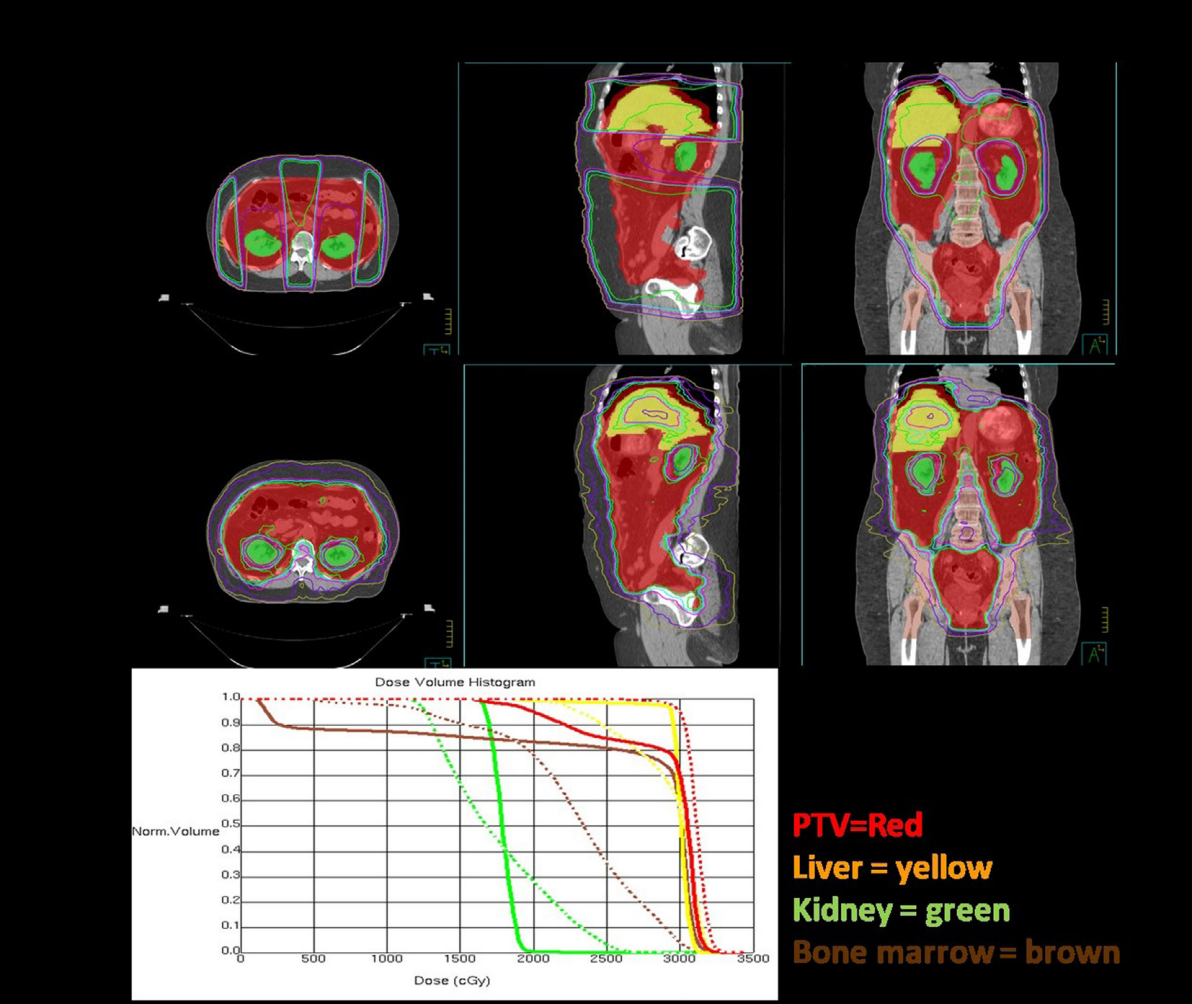
Dosimetric comparisons of three dimensional conformal and intensity modulation radiation therapy (IMRT) plans for whole abdominal radiotherapy. The plans are each shown in axial, sagittal, and coronal orientations and the dose volume histogram comparison is below. The planning target volume (whole abdominal cavity) is red, liver is yellow, kidneys are green, and bone marrow is brown. The IMRT plan is the dashed lines and the 3DCRT plan DVH’s are solid.

Figure 3 shows a comparison of an open-field three dimensional conformal radiotherapy plan with an intensity-modulated dynamic arc plan. The open-field plan is an AP-PA technique with a posterior kidney block to keep the mean dose to the kidneys <18 Gy. In order to encompass the large treatment volume, the open-field is treated at extended source to skin distance of 125 cm and the arc plan uses two isocenters with four arcs calculated on each. Arc techniques with helical tomotherapy, as used by Rochet et al. (74) are ideal as tomotherapy has a field width of 40 cm and length of 160 cm and the advantage of daily mega voltage CT setup.

On review of the dose volume histogram, the major differences between the two techniques is that, with the kidney blocks, there is a large dose gradient, which leads to dose heterogeneity with the three dimensional technique compared to superior coverage of the target volume with IMRT. With IMRT, the bone marrow and liver are able to be kept to lower doses, as shown in the sagittal and coronal images. The entire lung volume was not captured on this scan and, therefore, the volume receiving 20 Gy cannot be accurately calculated. However, IMRT does not significantly change the dose to the basal portions of the lungs and, therefore, would not be likely to reduce the risk of radiation pneumonitis. As one would expect with IMRT, a slightly higher volume of lung tissue receives a low dose and slightly lower volume receives a high dose or radiation, but the mean dose is similar. Increased conformality of IMRT comes at a cost, as it is much more labor intensive for the physician, dosimetrist, and physicist with many more contours, longer computer calculations, and more quality analysis. However, when planned, setup and treated carefully, the treatment is tolerable and potentially beneficial for the intermediate and high-risk groups of patients identified above.

### Low-Dose Fractionated Whole Abdominal Radiation As a Chemotherapy Sensitizer

In the modern era, low-dose fractionated radiotherapy to the upper abdomen has been demonstrated to be well tolerated in combination with chemotherapy in several disease sites. Radiation-induced DNA damage may potentiate the effects of drugs such as gemcitabine and other antimetabolites that inhibit DNA synthesis by incorporating into new DNA strands as they are synthesized. Other potential mechanisms by which radiation and chemotherapy may act synergistically include changes in the microenvironment such as decreased hypoxia, stabilized vasculature, and enhanced immune activation (77, 78).

Low-dose fractionated WAR was combined with gemcitabine in patients with pancreatic cancer with acceptable toxicity in a small, single institutional trial (79). This led to a multi-institutional phase I study combining low-dose WAR with gemcitabine and erlotanib in patients with pancreatic cancer in which treatment was well tolerated with encouraging efficacy (80). The GOG administered WAR (60 cGy × 2 fractions daily, days 1 and 4 of each week for 6 weeks) as a chemosensitizer for dose-escalated weekly paclitaxel in women with recurrent ovarian, fallopian tube, or peritoneal cancers (9). Three (30%) of 10 patients had stable disease for at least 6 months. Dose-limiting toxicities were primarily hematologic but also included one Grade 3 diarrhea.

Poly(ADP-ribose) polymerase (PARP) is an important family of enzymes activated in response to single-strand damage of DNA (81, 82). Increased PARP activity is a well-described mechanism by which tumor cells avoid apoptosis caused by DNA damaging agents; it has been linked to drug resistance and the ability of tumor cells to withstand genotoxic stress (83–85). PARP inhibitors interrupt the catalytic effects of PARP and have demonstrated activity particularly in cancers with defects in homologous recombination such as ovarian cancers with BRCA mutations and other markers of homologous recombination repair (82). Although cancer cells with defective homologous recombination (e.g., BRCA mutated or platinum sensitive) are noted to have enhanced sensitivity to PARP inhibition, homologous recombination deficiency is not always an accurate predictive biomarker of PARP inhibitor activity (86, 87). The DNA damage induced by radiation may destabilize DNA repair systems within the cancer cell, allowing for enhanced activity of PARP inhibition (87–89).

In a preclinical model of the combination of PARP inhibition and radiation, significant cell death *in vitro* was demonstrated as well as inhibition of tumor growth in a pancreatic cancer mouse xenograft model (90). WAR (60 cGy × 2 fractions daily, days 1 and 5 of each week for 3 weeks) was used as a chemosensitizer for dose-escalated twice-daily veliparib in patients with solid tumor malignancies associated with peritoneal carcinomatosis (91). Twelve (57%) achieved stable disease, with seven (33%) having stable disease for at least 6 months. Patients with gynecologic malignancy had the best responses and a platinum sensitive ovarian cancer patient with a germline BRCA mutation was an exceptional responder with a response of several years (91). A maximum tolerated dose of 250 mg BID was identified in an expansion cohort of ovarian cancer patients with overall reasonable toxicity (92). Clinical trial of PARP inhibitors plus radiation therapy are ongoing in breast cancer, rectal, head and neck and non-small cell lung cancer (Table 3). A proposed randomized Phase 2 trial of PARP inhibitor with or without radiation in ovarian cancer will provide additional insight into the role of the combination in this disease.

**Table 3:** Select ongoing clinical trials of radiation combinations.

Description	Phase	Disease site	NCT number	Agent(s)	Sponsor
**Poly(ADP-ribose) polymerase + radiation**					
Olaparib and Radiotherapy in head and neck cancer	I	Squamous cell carcinoma of the larynx stage II–III	NCT02229656	Olaparib 25–300 mg BID	The Netherlands Cancer Institute

Phase I study of olaparib combined with cisplatin-based chemoradiotherapy to treat locally advanced head and neck cancer (ORCA-2)	I	High-risk locally advanced HNSCC	NCT02308072	Olaparib 50–200 mg BID Cisplatin 35 mg/m^2^ Q week	Cancer Research UK

Olaparib and radiotherapy in inoperable breast cancer	I	Breast cancer or local recurrence of breast cancer, which is inoperable or/and metastatic, including inflammatory breast cancer	NCT02227082	Olaparib 25–400 mg BID	The Netherlands Cancer Institute

Veliparib with or without radiation therapy, carboplatin, and paclitaxel in patients with stage III non-small cell lung cancer that cannot be removed by surgery	I/II	Unresectable stage IIIA/IIIB, non-small cell lung cancer	NCT01386385	Arm I Carboplatin, Paclitaxel Arm II Carboplatin, Paclitaxel, Velaparib	NCI; Southwest Oncology Group

Veliparib and combination chemotherapy in treating patient with locally advanced rectal cancer	II	Locally advanced adenocarcinoma of the rectum, Stage II/III	NCT02921256	Arm I (mFOLFOX6, capecitabine) Arm II (mFOLFOX6, capecitabine, veliparib)	NCI; NRG Oncology

**Immunotherapy + radiation**					

FLT3 ligand immunotherapy and stereotactic radiotherapy for advanced non-small cell lung cancer	II	Stage III/IV non-small cell lung cancer not amenable to curative therapy	NCT02839265	FLT3 ligand therapy (CDX-301) with SBRT	Albert Einstein College of Medicine, Inc.

Checkpoint blockade immunotherapy combined with stereotactic body radiotherapy in advanced metastatic disease	II	Metastatic cancer with at least one lesion amenable to SBRT	NCT02843165	Checkpoint blockade immunotherapies (anti-CTLA-4 and anti-PD-1/PD-L1 antibodies) with SBRT	University of California, San Diego

ProstAtak^®^ Immunotherapy with standard radiation therapy for localized prostate cancer	III	Localized prostate cancer meeting the NCCN criteria of intermediate risk or patients having only one NCCN high-risk feature	NCT01436968	Arm I ProstAtak^®^(AdV-tk) + valacyclovir Arm II Placebo + valacyclovir	Advantagene, Inc.

Ipilimumab and stereotactic body radiotherapy (SBRT) in advanced solid tumors	I/II	Metastatic cancer with at least one metastatic or primary lesion in the liver, lung, or adrenal gland	NCT02239900	Ipilumumab with SBRT	M.D. Anderson Cancer Center; Bristol-Myers Squibb

Pembrolizumab and chemoradiation treatment for advanced cervical cancer	II	Locally advanced cervical cancer stage IB1 with lymph nodes or IB2–IVA	NCT02635360	Arm I Cisplatin-based chemoradiation with consolidative pembrolizumab × 3 cycles Arm II Chemoradiation with concurrent Pembrolizumab × 3 cycles	University of Virginia; Merck Sharp & Dohme Corp

Radiation can induce multiple forms of DNA damage, which is repaired within the cell by various methods. Defective non-homologous end joining (NHEJ) has been demonstrated in ovarian cancer cell lines (93). The inhibition of NHEJ leads to persistent DNA damage, which in turn leaves cells a more sensitive to radiation. Enhanced radiation response with inhibitors of NHEJ has been demonstrated in pancreatic cancer cell lines and may be rational in ovarian cancer as well (94).

### Abscopal Effect and Immunotherapy

Immunotherapy has revolutionized cancer care over the last few years. The immune environment is crucial to prognosis in ovarian cancer and efforts are underway to translate this to novel therapeutic applications (95). The potential contribution of the immune system in response to radiation therapy has been demonstrated—induction of tumor associated antigens have been shown to develop after radiation in colorectal and prostate cancers (96–98).

The term “abscopal” was originally used in 1953 to describe the systemic effects of radiation on “out-of-field” tumor deposits (99). An abscopal effect of radiation presumably works through the release of tumor antigens occurring when radiation exerts DNA damage to a tumor site and likely related to systemic secretion of specific cytokines and chemokines triggering a systemic immune response against local tumor antigens (100–102). This effect has been demonstrated in preclinical and animal models as well as reported in several case series (69, 103–105). Several proof of principal trials are ongoing to determine whether the abscopal effect can be augmented by administering radiation therapy in conjunction with immune activating agents (Table 3).

Preclinical evidence suggest that cell kill by radiation therapy activates immune responses of dendritic cells, CD4+ T lymphocytes, and CD8+ T lymphocytes (104, 106). Two mechanisms by which radiation has the potential to synergize with immunotherapy include (1) the generation of antigen-specific, adaptive immunity—act as sensitizer for anticancer agents—a phenomenon referred to as “*in situ”* vaccination, and (2) the induction of chemokine production to facilitate recruitment of effector T cells and reprogram the tumor microenvironment (103).

## Conclusion

Ovarian cancer has demonstrated sensitivity to radiation therapy. Toxicity in the historical setting has limited present day use of this treatment modality. However, an updated understanding of the molecular differences of distinct histologic subtypes of ovarian cancer with differential response to both chemotherapy and radiation therapy has generated renewed interest in the potential application of radiation therapy in ovarian cancer.

## Author Contributions

EF and ST participated in developing the concept of this manuscript, researching and writing, manuscript preparation, and approval of the final manuscript draft. WM and LL participated in researching and writing this manuscript, manuscript preparation, and approval of the final manuscript draft.

## Conflict of Interest Statement

The authors declare that the research was conducted in the absence of any commercial or financial relationships that could be construed as a potential conflict of interest.
